# Antimicrobial use and combination of resistance phenotypes in bacteraemic *Escherichia coli* in primary care: a study based on Japanese national data in 2018

**DOI:** 10.1093/jac/dkad379

**Published:** 2023-12-12

**Authors:** Yumiko Hosaka, Yuichi Muraki, Toshiki Kajihara, Sayoko Kawakami, Aki Hirabayashi, Masahiro Shimojima, Hiroki Ohge, Motoyuki Sugai, Koji Yahara

**Affiliations:** Antimicrobial Resistance Research Center, National Institute of Infectious Diseases, Tokyo, Japan; Department of Clinical Pharmacoepidemiology, Kyoto Pharmaceutical University, Kyoto, Japan; Antimicrobial Resistance Research Center, National Institute of Infectious Diseases, Tokyo, Japan; Antimicrobial Resistance Research Center, National Institute of Infectious Diseases, Tokyo, Japan; Antimicrobial Resistance Research Center, National Institute of Infectious Diseases, Tokyo, Japan; Department of Academics, SUGIYAMA-GEN Co., Ltd, Tokyo, Japan; Department of Infectious Diseases, Hiroshima University Hospital, Hiroshima, Japan; Antimicrobial Resistance Research Center, National Institute of Infectious Diseases, Tokyo, Japan; Antimicrobial Resistance Research Center, National Institute of Infectious Diseases, Tokyo, Japan

## Abstract

**Background:**

Antimicrobial use (AMU) in primary care is a contributing factor to the emergence of antimicrobial-resistant bacteria. We assessed the potential effects of AMU on the prevalence of a combination of resistance phenotypes in bacteraemic *Escherichia coli* in outpatient care settings between primary care facilities (‘clinics’) and hospitals.

**Methods:**

Population-weighted total AMU calculated from the national database was expressed as DDDs per 1000 inhabitants per day (DID). National data for all routine microbiological test results were exported from the databases of a major commercial clinical laboratory, including 16 484 clinics, and the Japan Nosocomial Infections Surveillance, including 1947 hospitals. AMU and the prevalence of combinations of resistance phenotypes in bacteraemic *E. coli* isolates were compared between clinics and hospitals.

**Results:**

The five most common bacteria isolated from patients with bacteraemia were the same in clinics, outpatient settings and inpatient settings in hospitals, with *E. coli* as the most frequent. Oral third-generation cephalosporins and fluoroquinolones were the top two AMU outpatient drugs, except for macrolides, and resulted in at least three times higher AMU in clinics than in hospitals. The percentage of *E. coli* isolates resistant to both drugs in clinics (18.7%) was 5.6% higher than that in hospitals (13.1%) (*P* < 10^−8^).

**Conclusions:**

Significant AMU, specifically of oral third-generation cephalosporins and fluoroquinolones, in clinics is associated with a higher prevalence of *E. coli* isolates resistant to both drugs. This study provides a basis for national interventions to reduce inappropriate AMU in primary care settings.

## Introduction

Antimicrobial resistance (AMR) is becoming one of the most pressing healthcare threats over the globe. A review in the UK estimated that 10 million deaths a year will be attributable to AMR by 2050 unless action is taken.^1^ To tackle AMR, the WHO global action plan for AMR in 2015 highlighted the importance of optimized antimicrobial use (AMU) and AMR surveillance as strategic objectives,^2^ which accounts for the reduction in the overuse or misuse of antimicrobials. In particular, the appropriate use of oral antimicrobials, which account for the majority of antimicrobial prescriptions, should be encouraged in every medical facility.

It has been proven that there is a positive relationship between AMU and the development of antimicrobial-resistant bacteria because a natural evolutionary response to antimicrobial exposure is a major factor inducing AMR.^3–6^ In the battle with AMR, demonstrating this relationship in primary care, where substantial antimicrobials are prescribed, has attracted global attention, irrespective of the country’s income level.^3,4,7–10^ In Japan, the Ministry of Health, Labour and Welfare (MHLW) launched the National Action Plan (NAP) on AMR (2016–20), published the Manual of Antimicrobial Stewardship in 2017, which was mainly targeted at primary care,^11^ and introduced financial incentives for appropriate outpatient AMU for paediatric patients in 2018 and for otorhinolaryngological patients in 2022.^12,13^ Although NAP-based national interventions have reduced oral AMU to some extent,^14^ their impact on AMR has not yet been assessed. National intervention through financial incentives to reduce AMU in primary care has been attempted in several countries, such as Denmark, Sweden and the UK,^15–18^ but the impact of these interventions on AMR at the national level has been longitudinally assessed only in the UK.^18^

To date, no studies have focused on the relationship between AMU and the prevalence of antimicrobial-resistant bacteria by comparing data from different outpatient settings based on national AMR surveillance data. In this study, we used three national surveillance datasets to address this situation. The Japan Surveillance of Antimicrobial Consumption (JSAC)^19^ based on the National Database of Health Insurance Claims and Specific Health Checkups (NDB) of Japan includes national surveillance data of AMU and was utilized to compare AMU between different types of facilities providing outpatient care [primary care settings (‘clinics’) and hospitals]. We utilized data from routine microbiological test results from a major commercial clinical laboratory that covered ‘clinics’ across Japan in 2018 as national AMR surveillance data from clinic patients. For national AMR surveillance data from patients visiting hospitals, we used comprehensive surveillance data collected in a national antimicrobial resistance surveillance programme—the Japan Nosocomial Infections Surveillance (JANIS)—in which all routine microbiological test results have been collected for all sample types from both symptomatic and asymptomatic patients from hundreds or thousands of participating hospitals since 2000.^20,21^

Using three types of national data, we assessed the potential effects of AMU on the prevalence of a combination of resistance phenotypes among *Escherichia coli* isolated from patients with bacteraemia at different medical facilities providing outpatient care.

## Materials and methods

### Study design and data source

We conducted a cross-sectional study using national surveillance data of AMU and microbiological testing data from a major commercial clinical laboratory database in 2018, as well as the national AMR surveillance system database.

Specifically, we used publicly accessible surveillance data of AMU from the JSAC database, based on data from the NDB managed by the MHLW.^19^ Further information about the NDB and JSAC is provided in the Supplementary text (available as Supplementary data at *JAC* Online). The AMU data in the JSAC datasheet were calculated based on antimicrobial volume (total prescribed drug quantity) and not on antimicrobial prescribing, as follows: after extracting total prescribed drug quantity data through health insurance from the NDB and converting them to gram data, they were adjusted for drug strength and stored as AMU in the JSAC datasheet.^19^ Regarding routes of administration, oral antimicrobial use accounted for more than 90% of total AMU, and around three-quarters of them were cephalosporins, macrolides and fluoroquinolones as shown in previous reports.^22,23^

As for medical facility type, the Medical Care Act in Japan defines facilities with 0–19 beds as ‘clinics’, and those with 20 beds or more as ‘hospitals’. Most patients requiring primary-care related services go to clinics, while many outpatients in hospitals need specialty care, such as cancer chemotherapy, postoperative follow-up and immunosuppressive therapy. The estimated number of outpatients who visited clinics accounted for 72.1% of those requiring outpatient care (4 233 000 of 5 874 900 outpatients) in the national survey.^24^

All microbiological test results for all specimens related to isolated bacteria in 2018 were extracted from a major commercial clinical laboratory database consisting of 16 484 primary care facilities to obtain clinical data. We extracted all inpatient and outpatient data fields for all specimens collected from 1947 hospitals across Japan between January and December 2018 from the JANIS database, covering both culture-positive and culture-negative test diagnostic results. Data from a major commercial clinical laboratory and the JANIS database were de-duplicated according to the standard method of the WHO Global Antimicrobial Resistance Surveillance System to select only the first isolate of a given bacterial species per patient per surveillance period per specimen type per infection origin stratification.^25^

In the summary of medical institutions and hospital reports by the MHLW in 2017, the number of clinics and hospitals was 101 471 and 8412, respectively.^26^ One major commercial clinical laboratory that provided the data in this study covered 16.2% (16 484 clinics) of all clinics across Japan. The proportion of hospitals that voluntarily participated in JANIS was 23.1% of the 8412 hospitals across Japan in 2018.^27^

The microbiological data were available at the patient level and aggregated at the national level, while the AMU data were available only at the prefecture level and aggregated at the national level, as detailed below.

### Data preparation and analysis of AMU

The JSAC datasheet stratified the AMU data by route of administration, age category, type of care, inpatient or outpatient settings, facility type based on the number of beds, and prefecture based on the NDB. We aggregated the AMU data across the strata to obtain a national value for each antimicrobial class classified according to the WHO Anatomical Therapeutic Chemical (ATC) system.^28^ As the JSAC database includes data on both medical and dental care, we extracted only the data pertaining to medical care. To calculate the population-weighted total use expressed as DDDs per 1000 inhabitants/day (DID), which is an indicator of the AMU recommended by WHO,^29^ we divided the national AMU by the DDD of each antimicrobial according to the WHO ATC/DDD index before adjusting for the Japanese population in 2018.^28^ In cases in which the dose and dosage forms of antimicrobials proposed by the WHO ATC/DDD index are not applicable in Japan, the Japanese DDD (JDDD) proposed by the JSAC operator was used instead of the DDD.^30^

After data preparation, we compared the national AMU of oral and parenteral drugs categorized into ATC third or fourth levels (chemical subgroup). Further information about the drugs is provided in the legend of Figure S1.

### Data preparation and analysis of microbiological data

After de-duplication of the microbiological testing results, we tabulated the data on bacteria isolated from blood specimens using an in-house Java program^31^ to compare the top five bacteria commonly isolated from blood specimens of patients visiting ‘clinics (with 0–19 beds)’, ‘outpatient settings in hospitals (with ≥20 beds)’ and ‘inpatient settings in hospitals (with ≥20 beds)’. The tabulated ‘clinics’ data included only outpatient data because it is known that inpatients account for only 1.1% of total clinic patients.^24^ Given that the leading bacteria causing nosocomial infections are generally different from those causing community-acquired infections, bacteria isolated in inpatient settings in hospitals are expected to differ from those in clinics and outpatient settings in hospitals. Using the in-house Java program,^31^ we tabulated the resistance profiles (i.e. combinations of susceptible and resistant results for specific antimicrobial drugs) of *E. coli* isolated most frequently from bacteraemic patients in both clinics and hospitals at the national level.

### Analysis of the AMU of treatment drugs for *E. coli* bacteraemia

We selected five antimicrobials (penicillin with extended spectrum, combinations of penicillins, including β-lactamase inhibitors (‘combinations of penicillins’), first-generation cephalosporins, third-generation cephalosporins and fluoroquinolones) as the treatment drugs for *E. coli* bacteraemia because urinary tract infection (UTI) was the primary source for 53% of episodes of patients with *E. coli* bacteraemia^32^ and they were first- or second-line drugs for the treatment of UTI recommended by the Japanese Association of Infectious Diseases and Japanese Society of Chemotherapy (JAID/JSC) guide for the clinical management of infectious diseases.^33^ We compared the oral and parenteral AMU of these drugs in the three different settings.

### Examining the combination of resistance phenotypes in *E. coli*

To examine the combination of resistance phenotypes (‘resistance profiles’) to multiple antimicrobials in *E. coli*, third-generation cephalosporins and fluoroquinolones were selected as the core set of antimicrobials because these drugs are the most frequently prescribed antimicrobials in Japan for the treatment of outpatients with UTI.^34^ We selected ceftriaxone as a representative third-generation cephalosporin to detect the emergence of resistance based on a previous Japanese study where ceftriaxone resistance was detected at a higher percentage in ESBL-producing *E. coli* than cefotaxime resistance (97.4% versus 96.1%).^35^ Levofloxacin was selected as a representative fluoroquinolone because the percentage of oral levofloxacin was the largest of the total oral fluoroquinolones consumed (54.3%).^23^

The CLSI guidelines were used to categorize each isolate as susceptible or resistant, based on the results of antimicrobial susceptibility testing (AST).^36^ The chi-squared test was performed to test whether there was a significant difference in the frequency of a specific resistance profile between ‘clinics’ and ‘hospitals’ using R (version 4.1.1).

### Ethics

The JSAC data are available to the public; therefore, the data can be freely used. Data from one major commercial clinical laboratory do not include any identifiable data. Patient identifiers were de-identified by each hospital before data submission to JANIS. Anonymous data stored in the JANIS database were exported and analysed following approval by the MHLW (approval number 0425–2), according to Article 32 of the Statistics Act.

## Results

The total value of DID of oral antimicrobials was 22.6, whereas that of parenteral antimicrobials was 2.2, indicating that oral antimicrobials made up 91.1% of total AMU. Among oral AMU, macrolides (DID 8.4) were the most abundant, followed by fluoroquinolones (5.1), third-generation cephalosporins (4.6) and tetracyclines (1.7) (Figure S1). ‘Combinations of penicillins’ made up the majority of parenteral AMU (0.94).

**Figure 1.**
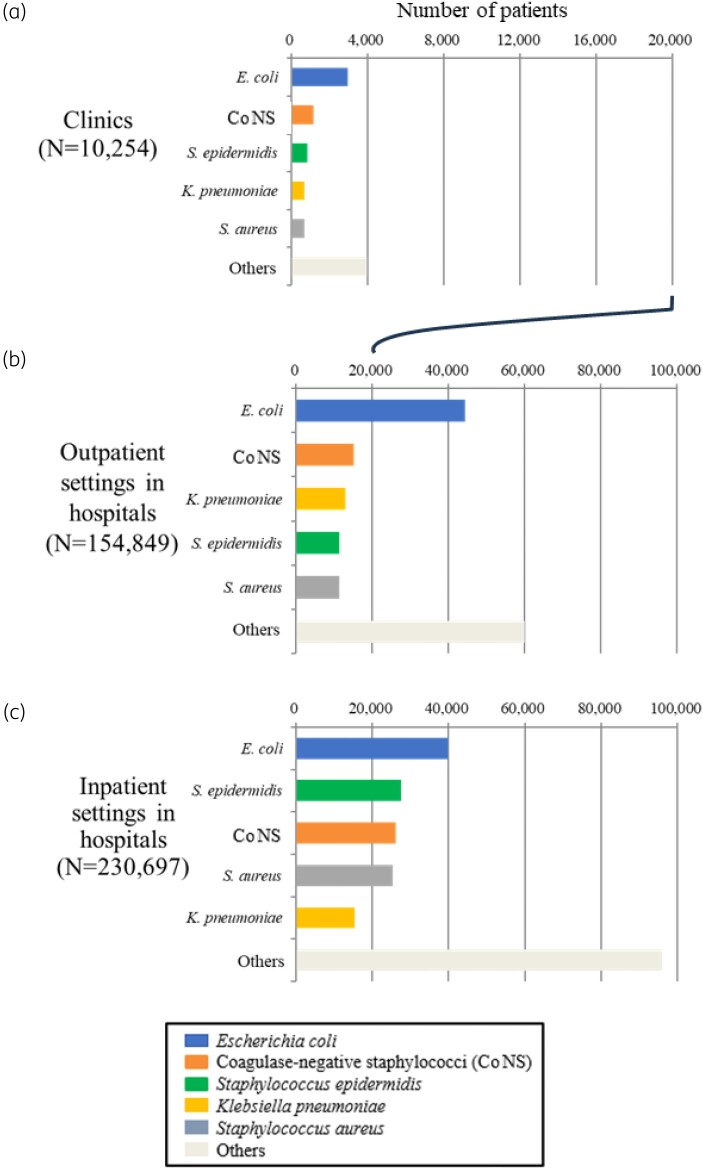
The top five bacteria commonly isolated from blood specimens of patients visiting three different settings. Each bar represents the number of patients from whom bacteria were isolated, including patients visiting (a) clinics, (b) outpatient settings in hospitals, and (c) inpatient settings in hospitals. The number of patients was counted without duplicates as mentioned in the Materials and methods section. Each differently coloured portion indicates one of the five isolated bacteria: *E. coli*, CoNS, *S. epidermidis*, *K. pneumoniae*, *S. aureus* and others.

Bacteria were isolated from blood specimens from 10 254 patients in clinics, 154 849 patients in outpatient hospital settings, and 230 697 patients in inpatient hospital settings (Figure 1). In all three settings, *E. coli* was the most frequently isolated bacterium from bacteraemic patients (Figure 1): the number of patients with *E. coli* bacteraemia without duplicates was 2963 (28.9%) and 44 301(28.6%) in clinics and outpatient settings in hospitals (Figure 1a and b), respectively, whereas it was 40 041 (17.4%) in inpatient settings in hospitals (Figure 1c). The five most common bacteria isolated from patients with bacteraemia (*E. coli*, CoNS, *Klebsiella pneumoniae*, *Staphylococcus epidermidis*, *Staphylococcus aureus*) were the same in clinics, outpatient settings and inpatient settings in hospitals. For further analyses, we focused on *E. coli* as the most frequently isolated species from patients with bacteraemia.

### Comparison of AMU of treatment drugs for *E. coli* bacteraemia

**Figure 2.**
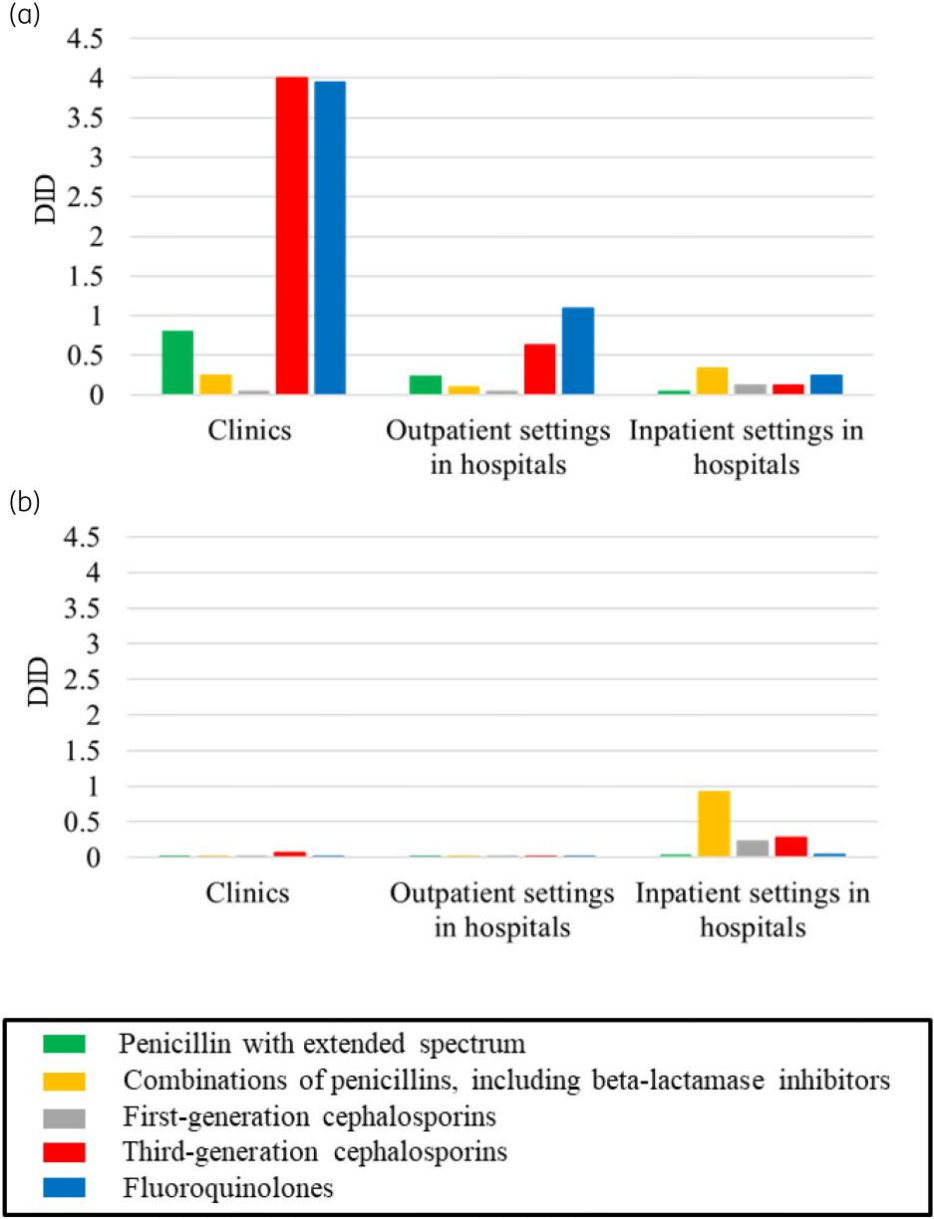
AMU (DID) of five types of oral antimicrobials among three different types of settings [clinics (0–19 beds), outpatient settings in hospitals (≥20 beds), and inpatient settings in hospitals (≥20 beds)] for (a) oral antimicrobials, (b) parenteral antimicrobials. Each bar indicates the DID value for one of the five groups of antimicrobials: penicillin with an extended spectrum, ‘combinations of penicillins, including β-lactamase inhibitors’, first-generation cephalosporins, third-generation cephalosporins and fluoroquinolones.

Figure 2 shows the AMU of five oral (a) and parenteral (b) antimicrobials in three different settings. Remarkably, the values of DID of oral third-generation cephalosporins and fluoroquinolones in clinics were 4.0 and 3.9, respectively, which were at least three times higher than values in hospitals (0.63 and 1.1, respectively, in outpatient settings and 0.12 and 0.24, respectively, in inpatient settings). Regarding the AMU of the other three drugs, penicillin with an extended spectrum (green in Figure 2a) exhibited a higher DID value (0.80) in clinics than in hospitals [0.24 (outpatient settings) and 0.037 (inpatient settings)], whereas the DID value of ‘combinations of penicillins’ (orange in Figure 2a) in inpatient settings in hospitals (0.33) was higher than that in the other two settings [0.24 (clinics) and 0.098 (outpatient settings, hospitals)].

In comparison, the DID values of the parenteral antimicrobials in all three settings were less than 1.0 (Figure 2b). The ‘combinations of penicillins’ AMU in inpatient settings in hospitals (0.92, orange in Figure 2b) was by far the largest of all three settings [0.0077 (clinics) and 0.0045 (outpatient settings in hospitals)]. The first- and third-generation cephalosporins AMU in hospital inpatient settings (0.23 and 0.28, respectively) was also higher than that in clinics (0.0054 and 0.067, respectively) and hospital outpatient settings (0.0039 and 0.019, respectively).

### Comparison of prevalence of combination of resistance phenotypes in *E. coli* bacteraemic patients

**Figure 3.**
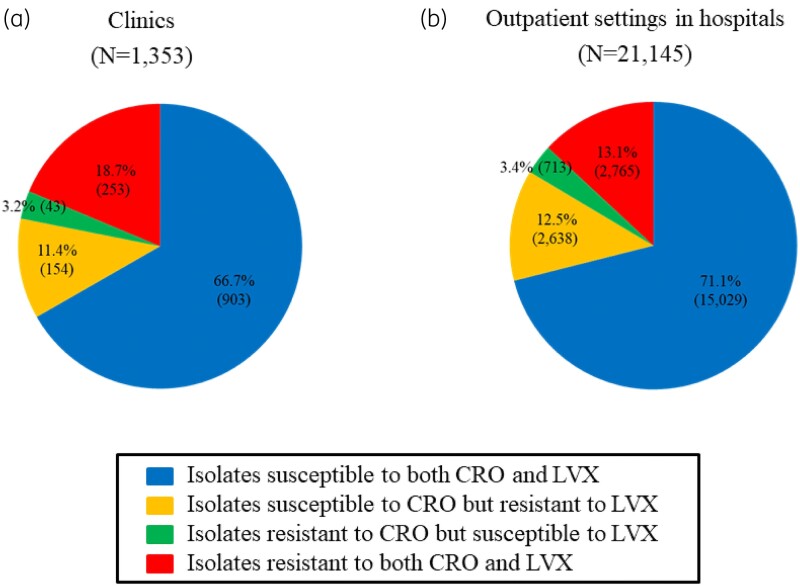
Pie charts of bacteraemic patients with *E. coli* isolates tested with two drugs [ceftriaxone (CRO) and levofloxacin (LVX)] categorized into four types according to the results of antimicrobial susceptibility to both drugs between (a) clinics (0–19 beds) and (b) outpatient settings in hospitals (≥20 beds) in 2018. The total number of bacteraemic *E. coli* patients with isolates tested with both drugs stratified by type of medical facility did not include those with isolates that were neither susceptible nor resistant to both drugs. The blue portion of the pie chart indicates the isolates that were susceptible to both CRO and LVX. The yellow portion represents isolates susceptible to CRO but resistant to LVX. The green portion indicates the isolates resistant to CRO but susceptible to LVX. The red portion represents isolates resistant to both CRO and LVX. The percentage of bacteraemic patients with each of the four types of *E. coli* isolates is shown in the pie chart along with the number of patients.

The number of bacteraemic *E. coli* patients with isolates tested with both ceftriaxone and levofloxacin was 1353 in clinics and 21 145 in outpatient settings in hospitals (Figure 3), after excluding those with isolates that were neither susceptible nor resistant to the two drugs. Among them, the percentage of patients with *E. coli* isolates resistant to both ceftriaxone and levofloxacin in clinics was 18.7% (253), which was 5.6% higher than that in outpatient settings in hospitals [13.1% (2765)] (*P* < 10^−8^, chi-squared test). Meanwhile, the percentage of patients with *E. coli* isolates susceptible to both ceftriaxone and levofloxacin in hospitals [71.1% (15 029)] was 4.4% higher than that in clinics [66.7% (903)] (*P* < 10^−3^, chi-squared test). As for the remaining two phenotypes (isolates resistant to ceftriaxone but susceptible to levofloxacin, as well as isolates susceptible to ceftriaxone but resistant to levofloxacin), the percentages of patients with *E. coli* isolates with distinct phenotypes were almost the same between clinics and hospitals [3.2% (43) and 3.4% (713), and 11.4% (154) and 12.5% (2638), respectively].

## Discussion

A notable finding of this study was the significant AMU of two major oral antimicrobials (third-generation cephalosporins and fluoroquinolones) for the treatment of UTI in outpatient clinics, which may contribute to the higher prevalence of bacteraemic *E. coli* isolates resistant to both drugs in clinics than in outpatient settings in hospitals in Japan. Most of these isolates are likely to be ST131, which is the major ST producing ESBL and resistant to quinolones^37,38^ and has contributed to the global spread and Japanese regional epidemic of ESBL-producing quinolone-resistant *E. coli*.^39,40^ Prescribing either oral third-generation cephalosporins or fluoroquinolones will increase their prevalence.

Given that clinics cover 72.1% of people requiring outpatient care and are responsible for primary care,^24^ promoting appropriate AMU in primary care is one of the keys to AMR containment in Japan. A previous study using the frequency of prescriptions revealed that a smaller facility scale was associated with higher odds of antimicrobial prescriptions for outpatients.^34^ This finding is consistent with the results of our study, showing that clinics are responsible for a substantial proportion of AMU in Japan through outpatient prescriptions. When bacterial species isolated in clinics from urine and respiratory specimens were examined, *E. coli* accounted for almost 50% of the total isolates from urine specimens, and four bacterial species (*Haemophilus influenzae*, *S. aureus*, *Moraxella* (*Branhamella*) *catarrhalis* and *Streptococcus pneumoniae*) constituted 59.3% of all isolates from respiratory specimens (Figure S2). Under the JAID/JSC guidelines, oral fluoroquinolones and third-generation cephalosporins are recommended as first- or second-line antimicrobials for outpatients with UTIs on the premise that *E. coli*, the most frequent causative pathogen of UTIs, is susceptible to these two drugs.^33^ These guidelines also recommend oral fluoroquinolones as second-line antimicrobials for isolates susceptible to all drugs or first-line antimicrobials for isolates resistant to narrow-spectrum antimicrobials to treat respiratory infections caused by the four most common bacterial species from respiratory specimens. The recommendations of the JAID/JSC guidelines may explain the significant clinical use of oral third-generation cephalosporins and fluoroquinolones. In addition, a Japanese study of outpatient prescriptions found that the rate of first-line antimicrobial prescriptions for infections for which antimicrobials were frequently prescribed was only 24%, and the majority of non-first-line antimicrobials were third-generation cephalosporins, quinolones and macrolides.^41^

This study also showed that the resistance rates of bacteraemic *E. coli* to ‘combinations of penicillins’ and trimethoprim/sulfamethoxazole, which have a relatively narrow spectrum and are potential alternative outpatient antimicrobials to third-generation cephalosporins and fluoroquinolones in the treatment of UTI patients, were both over 15% in clinics (Figure S3). The resistance rates of *E. coli* isolated from urine (Figure S4) exhibited consistent results (Spearman’s correlation coefficient 0.97). Thus, the appropriate use of antimicrobials in clinics is becoming increasingly important in Japan.

England, which has a national healthcare system similar to Japan, introduced antimicrobial stewardship interventions targeting primary care to control AMR because antimicrobial prescriptions in primary care account for more than 70% of the total prescriptions, most of which are deemed inappropriate.^18,42^ This national programme offering financial incentives for the reduction in antimicrobial prescribing and broad-spectrum antimicrobial prescribing has led to a decrease in AMU to a certain extent.^18,43,44^ One study found that this intervention had a smaller impact on the reduction of resistance in *E. coli* causing bacteraemia than on AMU, and the reductions were not sustained in the long term.^18^ Another report found that it had both positive and negative effects on urinary *E. coli* AMR in the short term.^44^ These results are consistent with the situation that conclusions of studies on the effect of national policy-mediated outpatient antimicrobial restrictions on reduction in AMR among *E. coli* in the short-term in Israel and the UK were mixed.^43,45,46^ To provide evidence in this field, our suggestion of using national surveillance data to examine the association between AMU and drug resistance is crucial for assessing the short- and long-term impacts of national interventions on AMR in Japan.

We examined the combination of resistance to multiple major antimicrobials for the treatment of UTIs in bacteraemic *E. coli* isolates based on national surveillance data. Recently, using this approach, we were able to track the emergence of resistance in *S. aureus* using JANIS data from our previous study.^31^ The value of reporting and analysing susceptibility through ‘full susceptibility profiles’ has been recently highlighted, compared with reporting on each of the antimicrobials of interest separately.^47^ Our research demonstrates that the analysis of ‘full susceptibility profiles’ also works well for *E. coli* to detect a combination of resistance phenotypes that show a notable increase in frequency.

Our study had several limitations. First, this study used only 1 year of data because of the limited availability of clinical data from a commercial laboratory service company. Additional studies that collect and compare data over several years are warranted to confirm our hypothesis of an association between antimicrobial overuse and an increase in the combination of resistance phenotypes in primary care. Second, to conduct a more robust analysis based on the only 1 year data, it is necessary to have a facility identifier (ID) that is shared among the AMU dataset, microbiological dataset in clinics, and that in hospitals. However, a shared facility ID was not available for the three datasets. Third, AMU as measured by the DDD may underestimate the actual use in paediatric patients and patients with renal impairment compared with other measures, such as the frequency of AMU.^41^ Fourth, the participation rate of hospitals with fewer than 200 beds in JANIS was much lower than that of hospitals with 500 beds and more (14% versus 80.0%). Thus, larger hospitals may have a greater impact on the findings related to the JANIS data than smaller hospitals.^20^ Fifth, the small number of patients with *E. coli* causing bacteraemia in the data from clinics may not be sufficient to detect more combinations of resistance phenotypes compared with the hospital data from the JANIS database. Given that fewer blood cultures are obtained in clinics than in hospitals, future studies collecting larger amounts of bacteraemic patient data from clinics are warranted. Sixth, molecular phylogenetic analysis of *E. coli* causing bacteraemia is required to confirm the genetic characteristics of isolates showing the combination of resistance phenotypes used in our study.

Despite these limitations, to the best of our knowledge, this is the first study to explore the association between AMU and specific resistance profiles isolated from patients with bacteraemia in outpatient settings by comparing national surveillance data related to hospitals and those of a major commercial clinical laboratory covering clinics across Japan.

### Conclusions

Our results demonstrate the association between the significant AMU, specifically of oral third-generation cephalosporins and fluoroquinolones, and the higher prevalence of *E. coli* isolates resistant to both drugs in primary care. This evidence may encourage national interventions to promote appropriate AMU in primary care settings. Our approach will also help assess the impact of the Japanese National Action Plan-based intervention on AMR containment and provide valuable evidence on the impact of national antimicrobial stewardship interventions on AMR globally.

## Supplementary Material

dkad379_Supplementary_Data

## Supplementary data

Supplementary text and Figures S1 to S4 are available as Supplementary data at *JAC* Online.
