# Supplementary material for: Antimicrobial use and combination of resistance phenotypes in bacteraemic Escherichia coli in primary care: a study based on Japanese national data in 2018
Source: J Antimicrob Chemother. 2023 Dec 12;79(2):312–9. doi: 10.1093/jac/dkad379 (PMC10832589; doi:10.1093/jac/dkad379)
Supplement: dkad379_Supplementary_Data [file dkad379_supplementary_data.docx]

**Supplementary text**

**Further information about the NDB and JSAC**

In Japan, almost all citizens are enrolled in the universal health coverage (UHC) insurance system, which includes the Employee Health Insurance System and National Health Insurance System. This health insurance system covers 70–90% of medical costs, depending on age and salary, when patients require medical care. The NDB comprises health insurance claims data, including those of medical and pharmacy claims, that have been recorded electronically since 2009. In 2020, 98.6% of all health insurance claims were electronically recorded in the NDB database ^1^. Because the UHC does not include data on the medical costs of people receiving public assistance (2 million people [1.6% of all Japanese citizens in 2011]) funded by the government ^2^, such data are missing from the NDB. Further, the NDB comprises only anonymized data, including information regarding medical facilities, patients, and medical care. Facilities without the required number of beds are categorized as “others” in the NDB. When we compared the AMU data between different facilities based on the JSAC database, facilities classified as “others” under facility data were excluded owing to a lack of detailed data.

The AMR Clinical Reference Center of the National Center for Global Health and Medicine operates the JSAC. The JSAC data were reported in agreement with research on the implementation of the AMR action plan subsidized by a research grant from the MHLW ^3^. The AMR Clinical Reference Center has also proposed an antimicrobial database that includes DDD based on the WHO ATC/DDD index [^4^](#_ENREF_24).

The drug list of the JSAC for AMU includes antimicrobials that are coded as ATC 2^nd^ level (therapeutic subgroup) “J01” (antibacterials for systemic use) and categorizes them into the ATC 5^th^ level (chemical substance) [^4^](#_ENREF_24).

**References**

1. Ministry of Health, Labour and Welfare. *Manual for people planning to use NDB.* https://www.mhlw.go.jp/content/12400000/001076618.pdf
2. Ikegami N, Yoo B-K, Hashimoto H *et al*. Japanese universal health coverage: evolution, achievements, and challenges*.* *Lancet* 2011; **378**: 1106-15. https://doi.org/10.1016/S0140-6736(11)60828-3
3. AMR Clinical Reference Center. *Surveillance based on data from the NDB*. https://amrcrc.ncgm.go.jp/surveillance/010/20181128172333.html
4. AMR Clinical Reference Center. *Antimicrobial Database.* https://amrcrc.ncgm.go.jp/surveillance/030/20181128172757.html


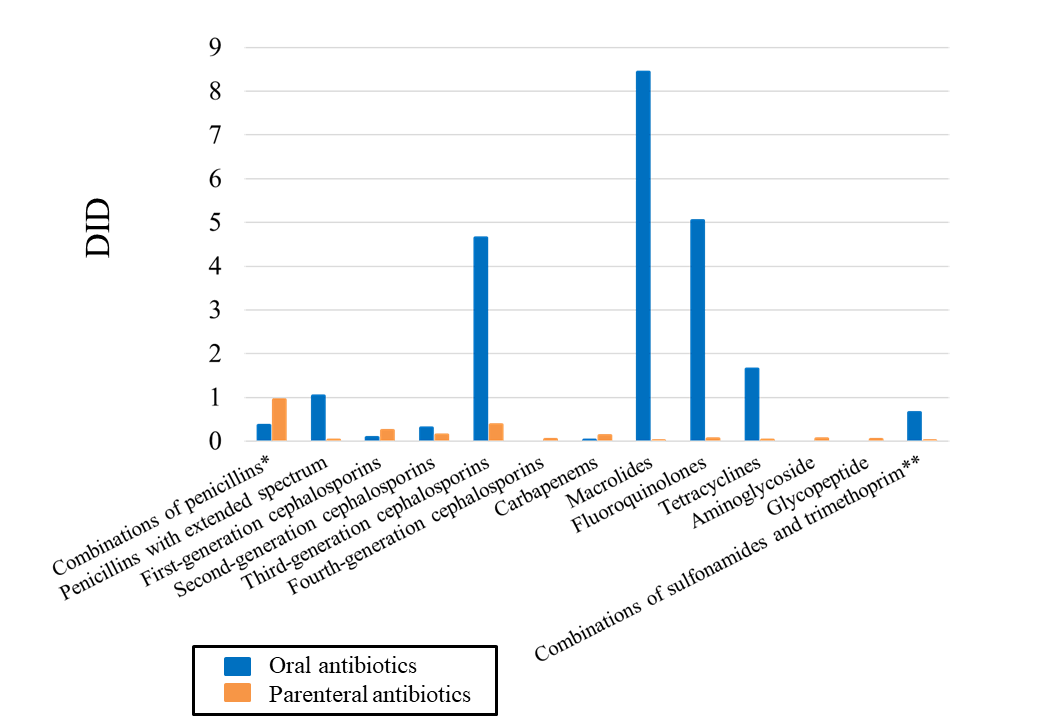


**Figure S1**. Consumption (DID [DDDs per 1,000 inhabitants/day]) of oral and parenteral antibiotics among ATC 3^rd^ or 4^th^ level (chemical subgroup) antibiotics. (J01A: tetracyclines; J01C: beta-lactam antibacterials; penicillins [J01CA: penicillins with extended spectrum: J01CR: combinations of penicillins, including beta-lactamase inhibitors]; J01D: other beta-lactam antibacterials [J01DB: first-generation cephalosporins; J01DC: second-generation cephalosporins; J01DD: third-generation cephalosporins; J01DE: fourth-generation cephalosporins; J01DH: carbapenems], J01E: sulphonamides and trimethoprim; J01F: macrolides, lincosamides, and streptogramins [J01FA: macrolides]; J01G: aminoglycoside antibacterials; J01M: quinolone antibiotics; J01X: other antibacterials [J01XA: glycopeptide antibacterials]).

Oral antibiotics are indicated by blue-colored bars, and parenteral antibiotics are indicated by orange-colored bars.

*Combinations of penicillins, including beta-lactamase inhibitors

**Combinations of sulphonamides and trimethoprim, including derivatives


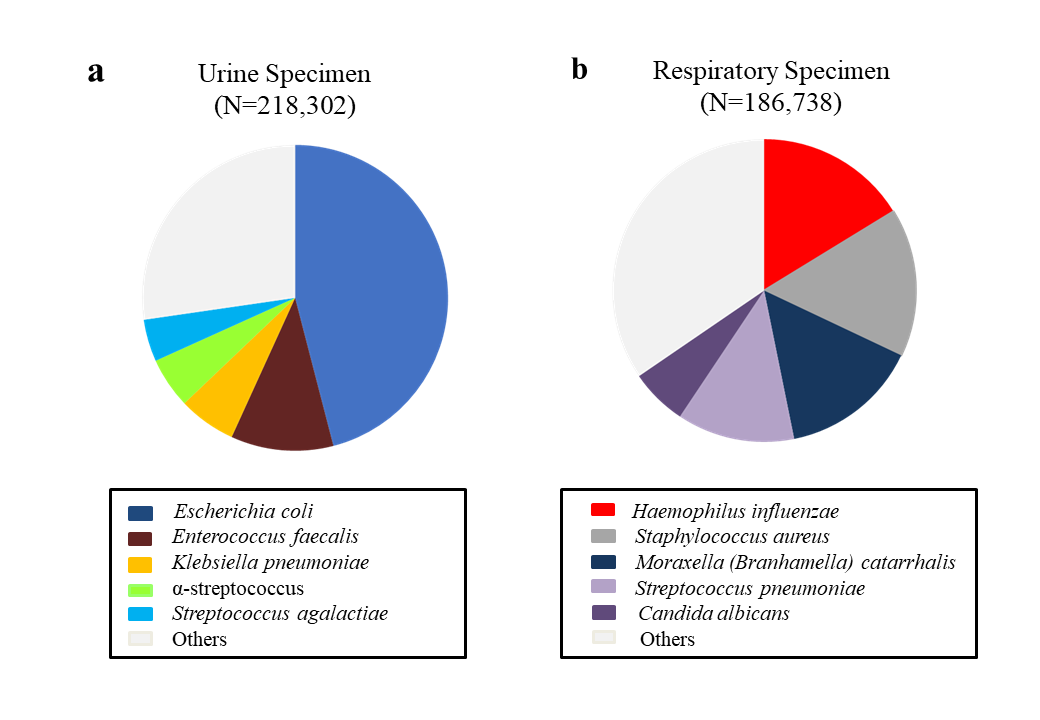


**Figure S2.** Pie charts of the top five bacteria commonly isolated from the urine and respiratory specimens of patients visiting clinics. Each pie chart represents the aggregated data of the number of patients from whom each bacterial strain was isolated from (a) urine and (b) respiratory specimens.

The total number of patients from whom bacteria were isolated is shown for each specimen type.

Each differently colored portion indicates the proportion of each of the five isolated bacteria: blue (*Escherichia coli*), brown (*Enterococcus faecalis*), light orange (*Klebsiella pneumoniae*), bright green (α-streptococcus), light blue (*Streptococcus agalactiae*), red (*Haemophilus influenzae*), grey (*Staphylococcus aureus*), dark grey blue (*Moraxella (Branhamella) catarrhalis*), light violet *(Streptococcus pneumoniae*), dark violet (*Candida albicans*), and light grey (Others).


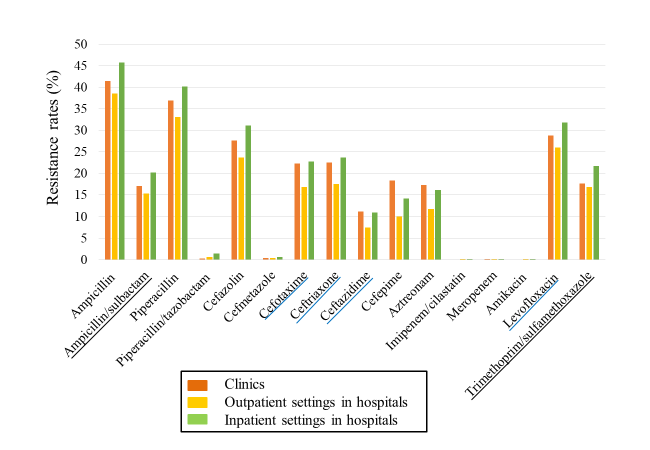


**Figure S3.** Resistance rates of *E. coli* isolated from bacteremic patients in three different settings (clinics, outpatient settings in hospitals, and inpatient settings in hospitals) to antibiotics categorized into ATC 3rd to 4th (chemical subgroup) levels recommended in the JAID/JSC guide as first- and second-line antibiotics for clinical management of infectious diseases, including urinary tract infections. Bars indicate the resistance rate of *E. coli* isolated from clinics (orange), outpatient settings in hospitals (yellow), or inpatient settings in hospitals (green).

Ampicillin/sulbactam and trimethoprim/sulfamethoxazole mentioned in the main text are highlighted in black. Third-generation cephalosporins and fluoroquinolones are indicated in blue.


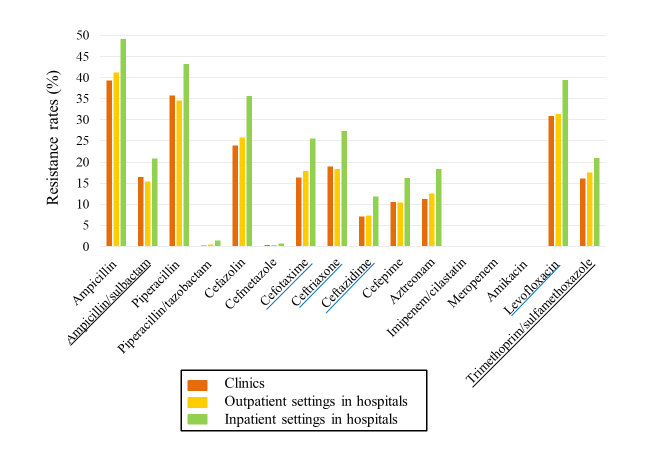


**Figure S4.** Resistance rates of *E. coli* isolated from urine specimen from patients visiting medical facilities between three different settings (clinics, outpatient settings in hospitals, inpatient settings in hospitals) regarding ATC3 to 4 level (chemical subgroup) antibiotics recommended in the JAID/JSC guide as first- and second-line antibiotics for clinical management of infectious diseases, including urinary tract infections. Each bar indicates the resistance rate of *E. coli* isolated in clinics (orange), outpatient settings in hospitals (yellow), and inpatient settings in hospitals (green), respectively.

Ampicillin/sulbactam and trimethoprim/sulfamethoxazole are underlined in black. Third-generation cephalosporins and fluoroquinolones are underlined in blue.
